# Prolonged duration of nonequilibrated Dirac fermions in neutral topological insulators

**DOI:** 10.1038/s41598-017-14308-w

**Published:** 2017-10-26

**Authors:** K. Sumida, Y. Ishida, S. Zhu, M. Ye, A. Pertsova, C. Triola, K. A. Kokh, O. E. Tereshchenko, A. V. Balatsky, S. Shin, A. Kimura

**Affiliations:** 1 Graduate School of Science, Hiroshima University, 1-3-1 Kagamiyama, Higashi-Hiroshima, Hiroshima 739-8526 Japan; 2 ISSP, University of Tokyo, 5-1-5, Kashiwa-no-ha, Chiba 277-8581 Japan; 3 State Key Laboratory of Functional Materials for Informatics, Shanghai Institute of Microsystem and Information Technology, Chinese Academy of Sciences, 865 Chang Ning Road Shanghai 200050, China; 4 Nordita, Roslagstullsbacken 23, SE-106 91, Stockholm, Sweden; 5 Center for Quantum Materials (CQM), KTH and Nordita, Stockholm, Sweden; 6 Institute of Geology and Mineralogy, Siberian Branch, Russian Academy of Sciences, Koptyuga pr. 3, 630090 Novosibirsk, Russia; 7 Novosibirsk State University, ul. Pirogova 2, 630090, Novosibirsk, Russia; 8 Saint Petersburg State University, Saint Petersburg, 198504 Russia; 9 Institute of Semiconductor Physics, Siberian Branch, Russian Academy of Sciences, pr. Akademika Lavrent’eva 13, 630090 Novosibirsk, Russia; 10 Institute for Materials Science, Los Alamos National Laboratory, Los Alamos, New Mexico, 87545 USA; 11 ETH Institute for Theoretical Studies, ETH Zurich, 8092 Zurich Switzerland; 12 Department of Physics, University of Connecticut, Storrs, CT 06269 USA

## Abstract

Topological insulators (TIs) possess spin-polarized Dirac fermions on their surface but their unique properties are often masked by residual carriers in the bulk. Recently, (Sb_1−*x*_Bi_*x*_)_2_Te_3_ was introduced as a non-metallic TI whose carrier type can be tuned from *n* to *p* across the charge neutrality point. By using time- and angle-resolved photoemission spectroscopy, we investigate the ultrafast carrier dynamics in the series of (Sb_1−*x*_Bi_*x*_)_2_Te_3_. The Dirac electronic recovery of ∼10 ps at most in the bulk-metallic regime elongated to >400 ps when the charge neutrality point was approached. The prolonged nonequilibration is attributed to the closeness of the Fermi level to the Dirac point and to the high insulation of the bulk. We also discuss the feasibility of observing excitonic instability of (Sb_1−*x*_Bi_*x*_)_2_Te_3_.

## Introduction

A three-dimensional (3D) topological insulator (TI) hosts spin-polarized massless Dirac states along its two-dimensional (2D) surface due to the topology of the bulk band structure^1–7^. The 2D metal on the surface exists as long as time reversal symmetry is preserved and exhibits novel phenomena not found in conventional 2D metals: These include the ballistic (high-mobility) transport due to the massless characteristic of the Dirac fermions and half-integer quantum Hall effects similar to those observed in graphene^8,9^.

While the surface of TIs is considered to be a promising platform for novel phenomena and applications, the surface-related properties are often masked by the residual carriers of bulk. Extensive efforts have been made to reduce the number of bulk carriers through the following guidelines^10–12^: (1) Design the band structures so that the Dirac bands are energetically isolated from the bulk bands; (2) Locate the Fermi level (*E*_F_) in the bulk band gap. Recently, it was shown that the carrier concentration can be controlled very precisely in the nonmetallic regime of the ternary TI (Sb_1−*x*_Bi_*x*_)_2_Te_3_^13–15^. Upon increasing *x*, the type of conduction changes from *p* to *n* across the intrinsic point. The bulk insulation is hallmarked by the demonstration of surface quantum Hall effect in a film sample^16^. (Sb_1−*x*_Bi_*x*_)_2_Te_3_ is thus regarded as a promising platform to unveil the exotic surface phenomena of TIs.

TIs irradiated by ultrashort light pulses have attracted much interest from both fundamental and application points of view. Angle-resolved photoemission spectroscopy (ARPES) implemented by the pump-probe method is a powerful tool to investigate the ultrafast phenomena of TIs. Time-resolved ARPES (TARPES) studies have disclosed novel states (Floquet^17^ and population-inverted states^18^), dynamics^19–23^, and functions (surface photovoltage (SPV) effect^24^ and generation of transient photocurrents^25^) on the nonequilibrated surface of TIs. Furthermore, it was recently proposed that these systems could host an excitonic insulating state particularly when the Dirac cone is neutrally doped^26^. Concerning the electronic recovery, there has been a dispute over the mechanism after the studies on bulk metallic TIs^27,28^. It is also not clear how the short ∼10-ps recovery for the bulk-metallic TIs^18–23^ is connected to the elongated ∼100-ps electronic recovery reported for bulk-insulating TIs^24,29^. In addition, it is not known whether or not there is a relationship between the electronic recovery and SPV effect, the latter observed in some bulk-insulating TIs^24,30^. Thus, a systematic study from the bulk-metallic to bulk-insulating regime is required for founding a solid platform to realize the novel light-induced phenomena.

In the present study, we investigate the ultrafastly-induced dynamics in a series of (Sb_1−*x*_ Bi_*x*_)_2_Te_3_ (*x* = 0, 0.29 and 0.43) by using TARPES. When the intrinsic point was approached, the electronic recovery time for the surface Dirac fermions was prolonged to >400 ps even though the SPV was not sizable. We thus attribute the prolonged duration to the increase of the bulk insulation and to the closeness of *E*_F_ to the Dirac point. We also discuss the feasibility of observing an excitonic instability for the near-neutral Dirac cone. The prolonged duration will facilitate the realization of novel optoelectronic functions such as optical gating of the high-mobility surface spin currents, efficient saturable absorption, and broad-band lasing^31^.

## Results

**Figure 1:**
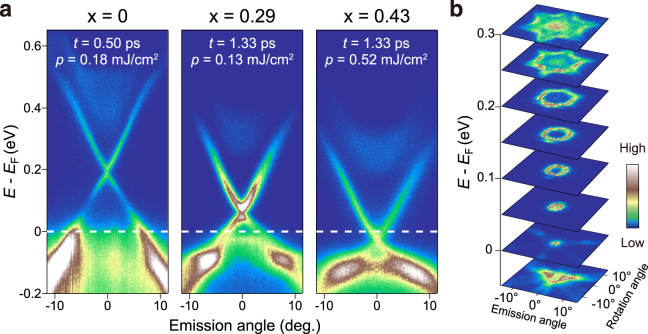
Band dispersions of (Sb_1−*x*_ Bi_*x*_)_2_Te_3_ (*x* = 0, 0.29, and 0.43). (**a**) Representative TARPES images displaying the bands in the unoccupied side. The cut is along $\overline{{\rm{\Gamma }}}-\overline{K}$ direction. (**b**) Constant energy maps of the angular distribution of photoelectrons for the *x* = 0.43 sample.

We start by showing the band dispersions of the samples *x* = 0, 0.29 and 0.43. In Fig. 1(a), we show typical TARPES images recorded at representative delay times *t* and pumping fluences *p*. All the images show the dispersions along the $\overline{{\rm{\Gamma }}}-\overline{K}$ line. The impact by the pump pulse redistributes the electrons across *E*_F_ and transiently fills the bands in the unoccupied side. Thus, the band structures above *E*_F_ can be accessed by TARPES. The Dirac cone dispersions and conduction bands above *E*_F_ are nicely observed. Upon increasing *x*, the Dirac point is shifted to lower energies. The Dirac points for *x* = 0, 0.29 and 0.43 are located at 190, 60 and −30 meV, respectively, showing that the filling of the Dirac cone changed from *p*- to *n*-type between *x* = 0.29 and 0.43. For *x* = 0 and 0.29, *E*_F_ crosses the valence band, while for *x* = 0.43, *E*_F_ is located within the bulk band gap; the results of which are consistent with previous ARPES studies^13^. Figure 1(b) shows constant energy maps of the angular distribution of photoelectrons for *x* = 0.43. Here, in order to reduce the matrix-element effect, the mapping was done by both *p*- and *s*-polarized probe, and the two maps were added. In going from the Dirac point to energies above *E*_F_, the distribution pattern gradually evolves from circular to hexagonal, due to the hexagonal warping effect that becomes pronounced at energies away from the Dirac point^32^.

**Figure 2:**
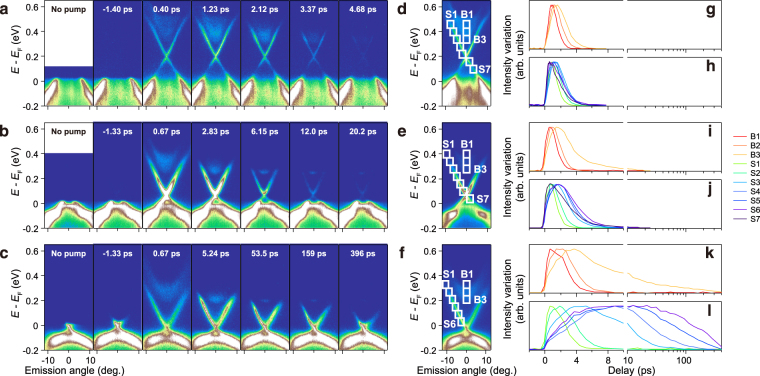
Nonequilibrium carrier dynamics in the (Sb_1−*x*_ Bi_*x*_)_2_Te_3_ crystals. (**a**–**c**) TARPES images recorded at various delay times for *x* = 0 (**a**), 0.29 (**b**) and 0.43 (**c**). (**d**–**f**) Frames set along the bands for *x* = 0 (**d**), 0.29 (**e**) and 0.43 (**f**). (**g**–**l**) Normalized intensity variations in the frames set in (**d**–**f**).

Figure 2(a)–(c) show TARPES images recorded at various pump-probe delay times for the three samples. We also display, in the left-most column, the images recorded in the absence of the pump pulse. In all cases, the unoccupied states are filled after the pumping at *t* = 0 ps. The main observation is that the time for the recovery from the nonequilibrated state elongates as *x* is increased: The filling is still observed at 396 ps in the case for *x* = 0.43, while for *x* = 0, the recovery is mostly accomplished already at 5 ps. Below, we look into the dynamics of the three samples one by one.

First, we focus on the sample *x* = 0 [Fig. 2(a)], whose Dirac point is located at the highest energy among the three. Before the arrival of the pump pulse (*t* = −1.4 ps), the photoemission signal is observed only below *E*_F_. The image is virtually unchanged from that recorded without the irradiation of the pump pulses [left-most panel of 2(a)], showing that the image recorded before *t* = 0 ps represents the spectrum of the equilibrium state. After the arrival of the pump pulse, for instance at *t* = 0.40 ps, the electrons are populated in the Dirac and bulk conduction bands located above *E*_F_. The excited state is gradually relaxed to the equilibrium state. We note that a novel population inversion across the Dirac point takes place during this period^18^. The relaxation is mostly accomplished by 5 ps. Next, we look into the dynamics of the sample *x* = 0.29 [Fig. 2(b)]. In this case, the Dirac point is located at an energy lower than that of *x* = 0. The image recorded at *t* < 0 ps represents that of the equilibrium state, as in the case for *x* = 0. At *t* = 0.67 ps, the bands in the unoccupied side are filled. During the recovery, some electrons remained at the bottom of the bulk conduction band; see the images recorded at *t* = 6.15 and 12.0 ps. The pump-induced variation is discerned for more than 20 ps. Finally, we describe the dynamics for the sample *x* = 0.43 [Fig. 2(c)], whose Dirac point is located closest to *E*_F_ among the three. Even at 400 ps, the excited electrons still remain not only in the Dirac band above *E*_F_ but also at the bottom of the bulk conduction band. We also notice that, unlike the *x* = 0 and *x* = 0.29 cases, the image recorded at *t* < 0 ps is shifted in energy from that recorded without pump, which we discuss later.

In order to resolve the energy-dependent carrier dynamics occurring in the bulk and surface bands, we set several frames along their dispersions as shown in Fig. 2(d)–(f), and plot, in Fig. 2(g)–(l), the intensity variations in each frame as a function of the pump-probe delay time. For the *x* = 0 sample [2(g) and 2(h)], the pump-induced variations are mostly diminished by 5 ps in all frames. For the *x* = 0.29 sample [2(i) and 2(j)], the recovery of the intensity takes longer than that of *x* = 0. It is also observed that delay in the filling (time until the maximum variation is reached) becomes prominent in the frames at lower energies. For the *x* = 0.43 sample [2(k) and 2(l)], whose Dirac point is located most closely to *E*_F_ among the three, the recovery time drastically elongates in going from the higher to lower energy frames in both the bulk [2(k)] and surface bands [2(l)]. The electrons remain at the bottom of the bulk conduction band (frame B3) even at 400 ps, as shown in Fig. 2(k). To our knowledge, such a long duration of the transient electrons at the conduction-band bottom has not been reported to date. In the surface bands [2(l)], the pump-induced variation persists for more than 100 ps in the frames located within the bulk band gap (S4 to S6). In particular, the duration exceeds 400 ps in the vicinity of *E*_F_ (S6). The delay in the filling also becomes pronounced in the *x* = 0.43 sample. In addition to the delayed filling, we also observe a time-resolution-limited rise in the intensity around *t* = 0 ps, which is notable in B3 and S3 to S6. This indicates that there are two types in the mechanism of the filling^33^; one that is similar to an impact ionization^18,34^, and the other that occurs through the transfer of electrons from high to low energies across the bands^19^.

**Figure 3:**
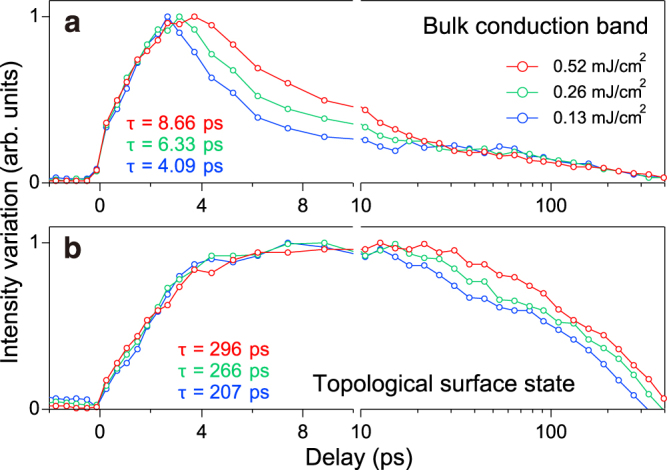
Pump-fluence dependency of the recovery time for *x* = 0.43. Intensity variations at the conduction-band bottom (B3) (**a**) and bottom of the upper Dirac cone (S6) (**b**) recorded at *p* = 0.13, 0.26 and 0.52 mJ/cm^2^.

In Fig. 3 we show the pump-fluence dependency recorded at *p* = 0.13, 0.26 and 0.52 mJ/cm^2^ for *x* = 0.43 sample. Figure 3(a) and (b) show the intensity variation and the recovery times at the bottom of the bulk conduction band (B3) and the bottom of the upper Dirac cone (S6), respectively. Upon increasing the pump fluence, both the bulk and surface recovery times are elongated. Even at *p* = 0.13 mJ/cm^2^, the fluence of which is comparable to those applied for *x* = 0 and 0.29 samples, the recovery time at S6 still exceeds 200 ps. The results strongly suggest that the prolonged recovery time of *x* = 0.43 sample is marginally affected by the fluence of the pump.

**Figure 4:**
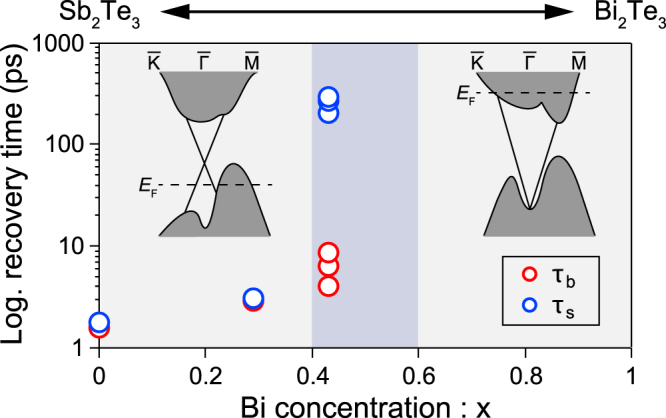
Recovery time recorded at various pumping fluences at the bottom of the bulk conduction-band (*τ*_*b*_) and the bottom of the upper Dirac cone (*τ*_*s*_) as functions of *x*.

We summarize the electronic recovery time in Fig. 4, in which we plot the electronic recovery time for bulk (*τ*_*b*_) and surface (*τ*_*s*_) taken at various pump fluence values as functions of *x*. Here, *τ*_*b*_ and *τ*_*s*_ were estimated respectively from the intensity variations in the frame at the bottom of the bulk conduction band and that located just above the Dirac point along the surface band^33^. By increasing *x* from 0 to 0.43, both *τ*_*b*_ and *τ*_*s*_ are increased. *τ*_*s*_ exceeds 300 ps for the bulk-insulating *x* = 0.43 sample. The recovery time hardly depended on *p*, while it elongated upon increasing temperature^33^, the implication of which will be discussed later. For *x* = 1, the recovery time is reported to be ∼50 ps^22^.

**Figure 5:**
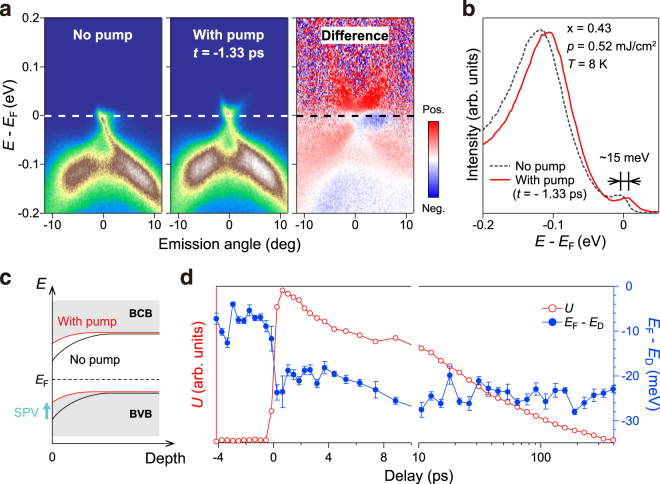
Surface photovoltage effect for the bulk insulating sample *x* = 0.43. (**a**) TARPES images recorded without (left) and with pump at *t* = −1.33 ps (middle panel). The right panel shows the difference image between two images. (**b**) Angle-integrated energy distribution curves for the images shown in (**a**, left and middle panels). (**c**) Schematic of the downward surface band bending and SPV effect. (**e**) Time-dependent electronic energy *U* (red color, left axis) and energy shift of the Dirac point (blue color, right axis).

We turn our attention to the photovoltaic shift of the spectrum observed for the *x* = 0.43 sample. The left and middle panels of Fig. 5(a) respectively display the TARPES images recorded without and with the irradiation of the pump pulses. The time delay was set to −1.33 ps for the latter. The right panel of Fig. 5(a) shows the difference between the two TARPES images (with pump - without pump). We can clearly see that the image is shifted upward in energy when the pump beam is irradiated. To emphasize the difference, Fig. 5(b) shows angle-integrated energy distribution curves of the images presented in the left and middle panels of Fig. 5(a). The shift upon the irradiation is estimated to be ∼15 meV, which is attributed to the emergence of SPV on the *x* = 0.43 sample. The SPV occurs when the insulation of the bulk is sufficiently high; then, an optically active band bending develops in the surface region [Fig. 5(c)]. The photovoltaic shift for the *x* = 0.43 sample thus fingerprints the insulating nature of the bulk and concomitant development of a downward surface band bending. The shift is observed even at *t* < 0 ps, because the duration of SPV exceeds the 4-*μ* s interval of the pump-probe events: That is, the SPV is maintained in the periodic steady state under the repetitive irradiation of the pump pulses at 250 kHz, and hence the shift is observable also at *t* < 0 ps^24,30^.

Finally, we investigate whether the SPV effect has some correlation with the electronic recovery. To this end, we plot in Fig. 5(d) as functions of the delay time, the locus of the Dirac point in energy and $U(t)\equiv {\int }_{\omega  > 0}\omega I(\omega ,t)d\omega$ where *ω* is the energy from *E*_F_ and *I*(*ω*, *t*) is the angle-integrated photoemission intensity^35^. The former represents the dynamics of SPV, and the latter represents the recovery of the electronic energy retained by the electrons above *E*_F_. The overall profile of *U*(*t*) shown in Fig. 5(d) nicely agrees with those shown in Fig. 2: The gradual recovery of the electronic excitation is seen to occur for ≳ 400 ps. On the other hand, the locus of the Dirac point is virtually unchanged in the time region [0, 400 ps] after the initial shift of −25 meV at *t* = 0 ps. The latter shows that the time for the SPV to recover is much longer than 400 ps: The SPV recovers for 25 meV with more than 4 *μ*s. Because the recovery time for the electronic excitation and that for SPV are orders of magnitude different, we consider that there is no correlation between the two.

## Discussion

Recently, a giant SPV shift of ∼50 meV was reported in a TARPES study of a bulk-insulating TI, Bi_2_Te_2_Se^24^. Even though the SPV shift therein is much larger than the ∼15 meV of the present case, the electronic recovery time of the surface state was as short as ∼100 ps^24^. The Dirac point for Bi_2_Te_2_Se was located at ∼300 meV below *E*_F_, which is deeper than the case for the *x* = 0.43 sample that exhibits the exceedingly long *τ*_*s*_ of ∼400 ps. Moreover, very recently, a large SPV (∼45 meV) was also observed in (Bi_0.45_Sb_0.55_)_2_Te_3_, whose the Dirac point is located at ∼150 meV below *E*_F_, at room temperature^36^. However, the electronic recovery time of surface state was as short as ∼25 ps. Based on these observations, we propose that: The prolonged electronic recovery of ∼400 ps observed for *x* = 0.43 is not only due to the bulk insulation but also due to the closeness of *E*_F_ to the Dirac point, while the size of the SPV is not directly related to the initial electronic recovery. Our view is supported by the fact that the electronic recovery time increased already from *x* = 0 to 0.29 even though the SPV effect was absent. When *E*_F_ approaches the Dirac point, the available phase space for the scattering within the Dirac cone (intra-band scattering) becomes small, so that the recovery of the Dirac electrons becomes less efficient. Besides, upon the increase in the bulk insulation, the photo-excited electrons in the Dirac cone will be less screened by the near *E*_F_ excitations in the bulk, which also contributes to the extension of the electronic recovery time within the Dirac cone.

Let us also discuss the possibility whether the exciton condensation can be formed in the optically excited state^26^. The formation of the excitonic insulating state is closely related to the dimensionless coupling constant α = *e* ^2^/*εħv* where *e* is the charge of the electron, *ε* is the system-specific dielectric constant, *ħ* is the Planck constant, and *v* is the Dirac velocity. When *α* is greater than the critical value (${\alpha }_{c}\approx 1$), it is expected that the electron-hole pairs formed by a mutual Coulomb attraction condense at low temperature^37–39^. Optically-excited Dirac materials are predicted to be a good candidate to realize the excitonic insulator. However, in 3D TIs for example Sb_2_Te_3_, *ε* is quite large (∼600), although *v* is small (∼2.3 × 10^5^ *m*/*s*) compared to graphene and other TIs^18,40^. This leads to a predicted excitonic gap less than 1 meV, which is much smaller than our energy resolution, and corresponds to a critical temperature that is quite low. The near-neutral Dirac cone nevertheless provides an interesting platform for the realization of the excitonic insulating state, if the material parameters are properly set.

## Methods

High quality (Sb_1−*x*_ Bi_*x*_)_2_Te_3_ single crystals of *x* = 0, 0.29, and 0.43 were grown by the Bridgman method^41^. Atomic ratios of the constituents were determined by using electron probe micro analysis. TARPES spectrometer consisted of a hemispherical electron analyzer and a Ti:sapphire laser system that delivered 1.48-eV pump and 5.92-eV probe pulses at the repetition rate of 250 kHz^42^. The pump and probe pulses had spot diameters of 280 and 85 *μ*m, respectively, on the sample. When recording the dynamics over 500 ps by varying the delay stage, it becomes crucial to keep the spatial overlap of the pump and probe beams throughout. We calibrated and checked that the spot of the pump beam on sample did not move for 5 *μ*m when the delay stage was shifted for 600 ps. This was done by using a pin hole of 200 *μ* m attached next to the sample. We set the locus of the pin hole at the focal point of the electron lens of the analyzer, and monitored that the power of the pump beam passing through the hole during shifting the delay stage. The energy and time resolutions were 15 meV and 300 fs, respectively. Samples were cleaved in the spectrometer at the base pressure 1 × 10^−8^ Pa, and were held at 8 K during the TARPES measurements. Spectral broadenings due to space charge effects were set to ≲10 meV.

## Electronic supplementary material

Supplementary
