# Supplementary material for: Prolonged duration of nonequilibrated Dirac fermions in neutral topological insulators
Source: Sci Rep. 2017 Oct 26;7:14080. doi: 10.1038/s41598-017-14308-w (PMC5658381; doi:10.1038/s41598-017-14308-w)
Supplement: Supplementary file 1 — Supplementary [file 41598_2017_14308_MOESM1_ESM.pdf]

Supplemental Material : Prolonged duration of nonequilibrated Dirac fermions in neutral topological insulators

K. Sumida[1,*], Y. Ishida[2,**], S. Zhu[1], M. Ye[3], A. Pertsova[4,5], C. Triola[4,5], K. A. Kokh[6,7,8], O. E. Tereshchenko[7,8,9], A. V. Balatsky[4,5,10,11], S. Shin[2] & A. Kimura[1,***]

[1]*Graduate School of Science, Hiroshima University, 1-3-1 Kagamiyama, Higashi-Hiroshima, Hiroshima 739-8526, Japan*

[2]*Institute for Solid State Physics, the University of Tokyo, 5-1-5, Kashiwa-no-ha, Chiba 277-8581, Japan*

[3]*State Key Laboratory of Functional Materials for Informatics, Shanghai, Institute of Microsystem and Information Technology, Chinese Academy of Sciences, 865 Chang Ning Road, Shanghai 200050, China*

[4]*Nordita, Roslagstullsbacken 23, SE-106 91 Stockholm, Sweden*

[5]*Center for Quantum Materials (CQM), KTH and Nordita, Stockholm, Sweden*

[6]*Institute of Geology and Mineralogy, Siberian Branch, Russian Academy of Sciences, Koptyuga pr. 3, 630090 Novosibirsk, Russia*

[7]*Novosibirsk State University, ul. Pirogova 2, 630090 Novosibirsk, Russia*

[8]*Saint Petersburg State University, Saint Petersburg, 198504, Russia*

[9]*Institute of Semiconductor Physics, Siberian Branch, Russian Academy of Sciences, pr. Akademika Lavrent'eva 13, 630090 Novosibirsk, Russia*

[10]*Institute for Materials Science, Los Alamos National Laboratory, Los Alamos New Mexico 87545, USA*

[11]*ETH Institute for Theoretical Studies, ETH Zurich, 8092 Zurich, Switzerland*

[*]e-mail : sumida1126@hiroshima-u.ac.jp [**]e-mail : ishiday@issp.u-tokyo.ac.jp

[***]e-mail : akiok@hiroshima-u.ac.jp

I. BULK AND SURFACE ELECTRONIC RECOVERY TIME

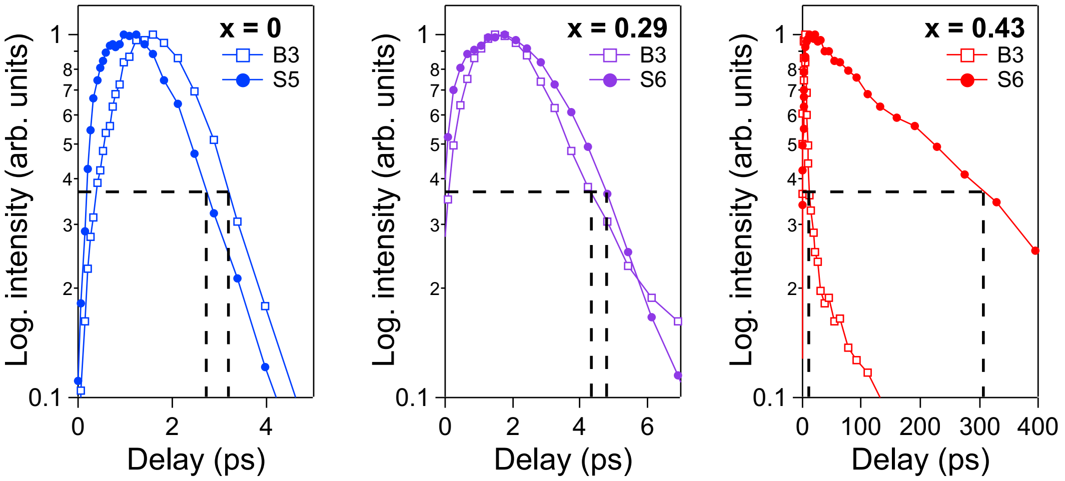

FIG. S1: Logarithmic-scale intensity variations for $x = 0$ (left), $x = 0.29$ (middle) and $x = 0.43$ (right panel) in the frame at the bottom of the bulk conduction band and the bottom of upper Dirac cone shown in Fig. 2(d - f) of the main text.

Figure S1 shows the logarithmic-scale intensity variations as a function of the pump-probe delay time for $x = 0$ (left), $x = 0.29$ (middle) and $x = 0.43$ (right panel). The energy-momentum frames were selected at the bottom of the bulk conduction band ($x = 0$: B3, $x = 0.29$: B3, $x = 0.43$: B3) and the bottom of the upper Dirac cone ($x = 0$: S5, $x = 0.29$: S6, $x = 0.43$: S6). The recovery time for bulk (τ_b) and surface (τ_s) were respectively estimated as a time required for an exponentially decreasing variable to drop from the maximum value to 1/e, as shown the dashed lines in Fig.S1. Table S1 summarizes τ_b and τ_s as functions of Bi concentration x.

TABLE S1: Recovery time at the conduction band bottom and the bottom of the upper Dirac cone.

	$x = 0$	$x = 0.29$	$x = 0.43$
τ_b (ps)	1.60	2.88	8.66
τ_s (ps)	1.76	3.05	296

II. SURFACE PHOTOVOLTAGE EFFECT

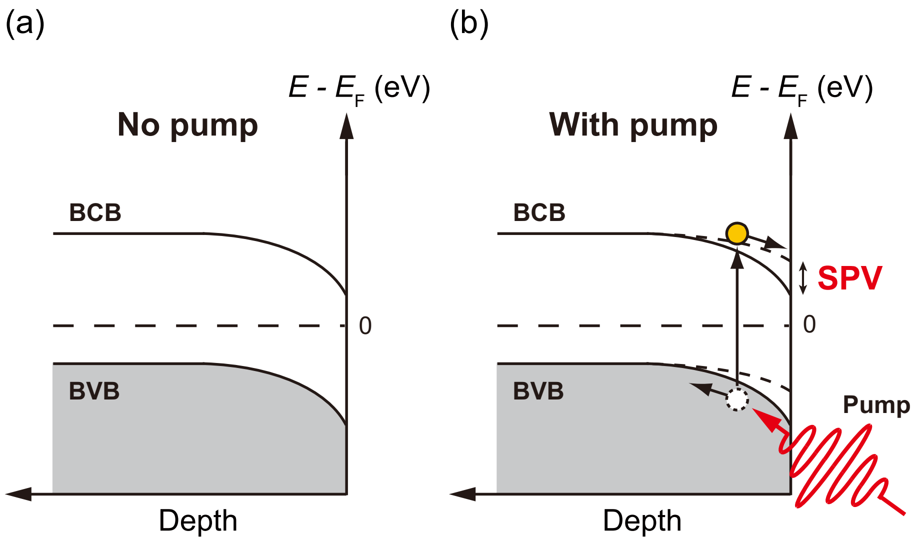

FIG. S2: Schematic images of downward band bending in a bulk insulating topological insulator without (a) and with the irradiation of the pump pulse (b).

We have observed a clear pump-induced chemical potential shift for the bulk insulating $x = 0.43$ sample [see : main text Figs. 5(a) and 5(b)]. This shift is attributed to the emergence of surface photovoltage (SPV) effect. SPV occurs when the insulating of the bulk is sufficiently high and optically active band bending develops in the surface region. Figure S2(a) shows the schematic image of the downward band bending in a bulk insulating topological insulator. Since the bulk bands are pinned to the Fermi level of the surface state, the band bending takes place at the interface. In such a case, by irradiation of the pump pulse, the electron-hole pairs are generated as shown in Figure S2(b). Because of the band bending, the generated electrons and holes drift in different directions. They are finally recombined in spatially different region but it takes longer time than other decay processes.

Figure S3 shows the Fermi cutoff of the Au and three samples. There are small space charge shift of the spectrum of 10 meV due to strong probing laser power in $x = 0$ and 0.29 samples compared with Au Fermi edge recorded at reduced probing power. However, that the difference between the spectra recorded without the pump laser irradiation and with the irradiation with the delay time of -1 ps is negligibly small.

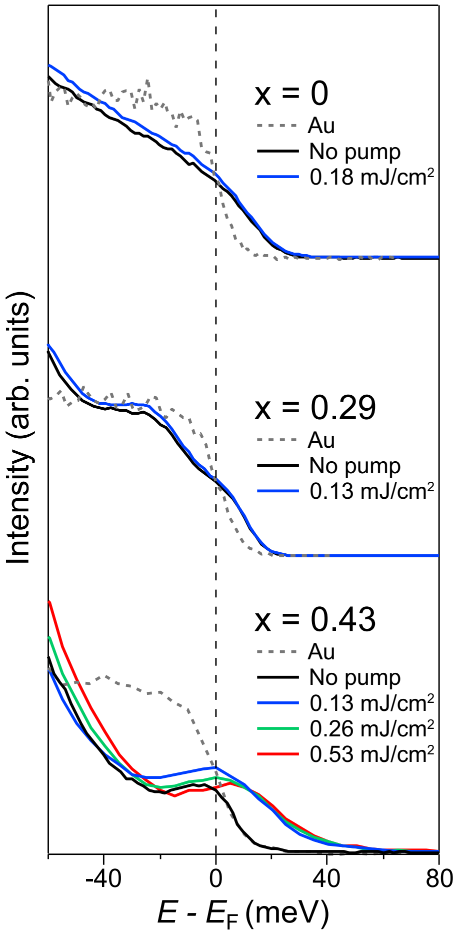

FIG. S3: Fermi cutoff of the Au and three samples recorded at t = -1 ps with pump and without pump.

Thus, this definitely shows that no SPV effect occurs in these samples. In contrast, for x = 0.43 sample, we can see obvious pump-induced shift in spite of the small space charge effect.

In addition, the carrier diffusion length of a typical semiconductor such as silicon is known to be on the order of micrometers. Since typical diffusion length is smaller than the diameter of probe pulse (85 micrometer), the electrons and holes are mostly recombined inside the illuminated area.

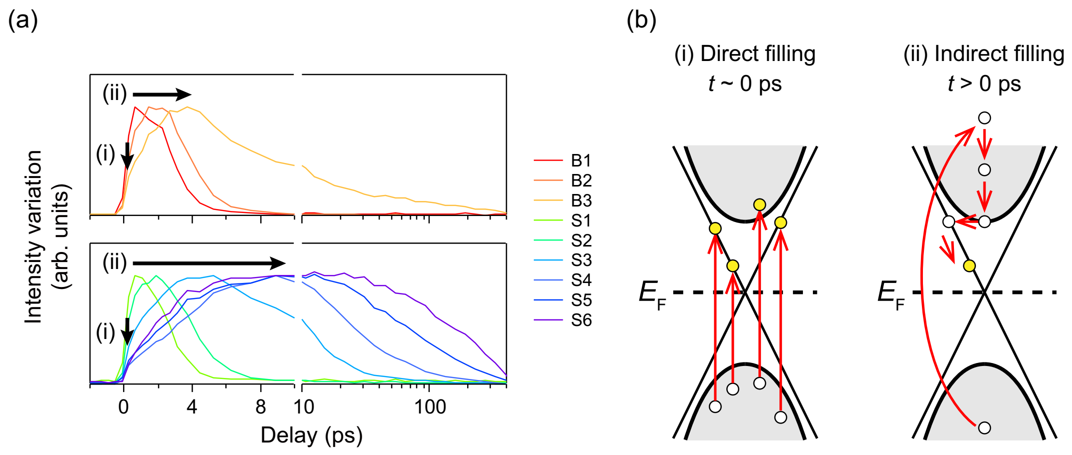

FIG. S4: Filling mechanism. (a) Normalized bulk (*upper*) and surface (*lower*) intensity variations for $x = 0.43$ in the frames set in main text Fig. 2(f). (b) Schematic images of two different filling mechanism; (b-i) Direct filling, (b-ii) Indirect filling.

III. FILLING MECHANISM

In Figure S4(a), we present again the intensity variations for $x = 0.43$ in each frame as a function of the pump-probe delay time. We can see two different filling mechanisms depicted in Fig. S4(a) as (i) and (ii). At pump-probe delay time t $\sim$ 0, the electrons are immediately populated to the upper states in the unoccupied region in two ways. The one is attributed to the impact ionization that occurs only in the vicinity of E_F [Fig. S4(b-i)]. The other is the indirect transition by intra-band and inter-band scatterings [Fig. S4(b-ii)]. The pump pulse excites the electrons to the higher energy states across the E_F. However the excited electrons are rapidly transferred to the lower-lying state via intra-band scattering [1]. In addition, some electrons remain at the bottom of the bulk conduction band, which acts as a reservoir of electrons that would make a radiative decay to the surface state [2]. As a result, we can see some delayed response in the several lower energy frames.

[1] J. A. Sobota, S. Yang, J. G. Analytis, Y. L. Chen, I. R. Fisher, P. S. Kirchmann, and Z.-X. Shen, Ultrafast Optical Excitation of a Persistent Surface-State Population in the Topological Insulator Bi_2Se_3, Phys. Rev. Lett. **108**, 117403 (2012).

[2] M. Hajlaoui, E. Papalazarou, J. Mauchain, G. Lantz, N. Moisan, D. Boschetto, Z. Jiang, I. Miotkowski, Y. P. Chen, A. Taleb-Ibrahimi, L. Perfetti, and M. Marsi, Ultrafast Surface Carrier Dynamics in the Topological Insulator Bi_2Te_3, Nano Lett. **12**, 3532 (2012).
